# Photoelectrochemical High-Value-Added Chemical Production with Improved Selectivity

**DOI:** 10.34133/research.0557

**Published:** 2024-12-23

**Authors:** Yingzhe Li, Tao Chen, Yihuang Chen

**Affiliations:** ^1^ Hubei Key Laboratory of Radiation Chemistry and Functional Materials, Hubei University of Science and Technology, Xianning, Hubei 437100, China.; ^2^ State Key Laboratory of Geological Processes and Mineral Resources, China University of Geosciences, Wuhan 430074, China.; ^3^ College of Chemistry and Materials Engineering, Wenzhou University, Wenzhou, Zhejiang 325035, China.

## Abstract

Photoelectrochemistry provides an important application in the production of high-value-added chemicals. However, photoelectrochemical organic transformation with high product selectivity remains a challenge. Until now, various technologies have been developed to promote the selectivity of photoelectrochemical high-value-added chemical production. Herein, a novel ion-shielding heterogeneous photoelectrocatalysis strategy for the production of trifluoromethyl group (CF_3_)-containing compounds with high selectivity is described.

To date, a variety of organic transformations have been achieved by electrochemically driven oxidation, which needs a large amount of electrical power. In contrast, photoelectrochemical (PEC) technology is highly attractive due to its reduced energy consumption with solar light assistance [1]. Nevertheless, undesired by-products are generated on the photoanodes due to the robust oxidizing capability of photoanodes and overoxidation of products, leading to a low product selectivity [2]. Thus, the development of PEC-selective oxidation of small molecules to target high-value-added chemicals is a highly demanding undertaking.

Trifluoromethyl group (CF_3_)-containing compounds are important in pharmaceuticals, agrochemicals, and organic materials due to their unique electronic structure and reactivity. As a consequence, the incorporation of CF_3_ into organic molecules has received intensive attention, as naturally occurring CF_3_-containing molecules are rare [3]. A traditional method for trifluoromethylation is metal-catalyzed direct C–H trifluoromethylation, mostly dominated by expensive trifluoromethylating reagents. Recently, trifluoroacetate salts and trifluoroacetic acid (TFA) have emerged as promising sources of trifluoromethyl groups (CF_3_) for their abundant and cost-effective properties [4]. In spite of remarkable advances, this decarboxylative pathway is hampered by the high oxidation potential of CF_3_COO^−^ (2.28 V versus a saturated calomel electrode), which needs harsh reaction conditions, such as stoichiometric amounts of external oxidants and highly oxidizing electrochemical conditions. More recently, in a *Science* paper, Chen et al. [5] from Zhejiang University realized highly efficient trifluoromethylation of (hetero)arenes via ion-shielding heterogeneous photoelectrocatalysis (IonShield-hPEC) under mild conditions. During the PEC trifluoromethylation of mesitylene, TFA is oxidized by photogenerated holes in the valence band of WO_3_ to generate the trifluoromethyl radical via decarboxylation. The trifluoromethyl reacts with the arene substrate to produce the trifluoromethyl arene radical, which is converted to the desired trifluoromethylated arene through a single-electron transfer process and subsequent deprotonation.

More importantly, negatively charged CF_3_COO^−^ was absorbed on the surface of WO_3_ photoanodes via electrostatic attraction, forming an ion-shielding layer. The absorbed CF_3_COO^−^ restricted the direct contact between the photoanode and the easier-to-oxidize mesitylene and thus prevented undesired mesitylene oxidation (Figure). During the PEC process, the current density did not show obvious change along with the increase in the concentration of the substrate mesitylene, confirming that the oxidation of CF_3_COO^−^ was favored over that of mesitylene under the IonShield-hPEC system. In contrast, mesityl trifluoroacetate was the main product via the direct oxidation of mesitylene without an ion-shielding layer due to its low oxidation potential (1.88 V versus a saturated calomel electrode). To improve PEC performance, Mo was doped into the WO_3_ photoanode in the range of 0.5% to 3.0% (Figure). After modification, over 69% yield of trifluoromethylated product was detected under mild conditions. Moreover, the stability of photoanodes is also important to PEC performance. In order to improve the stability of photoanodes, HCl was replaced by TFA during the preparation of Mo-doped WO_3_ photoanodes via the hydrothermal method. The photocurrent density of Mo-doped WO_3_ photoanodes remained a constant value throughout an around 380-h reaction with acceptable yield after modification (Figure). Furthermore, the original activity could be restored after reloading the used fluorinated tin oxide with new photoanodes, leading to a substantial reduction in the cost of photoanodes. Ultimately, large-scale PEC trifluoromethylation was achieved by numbering up modular photoanodes.

**Figure.**
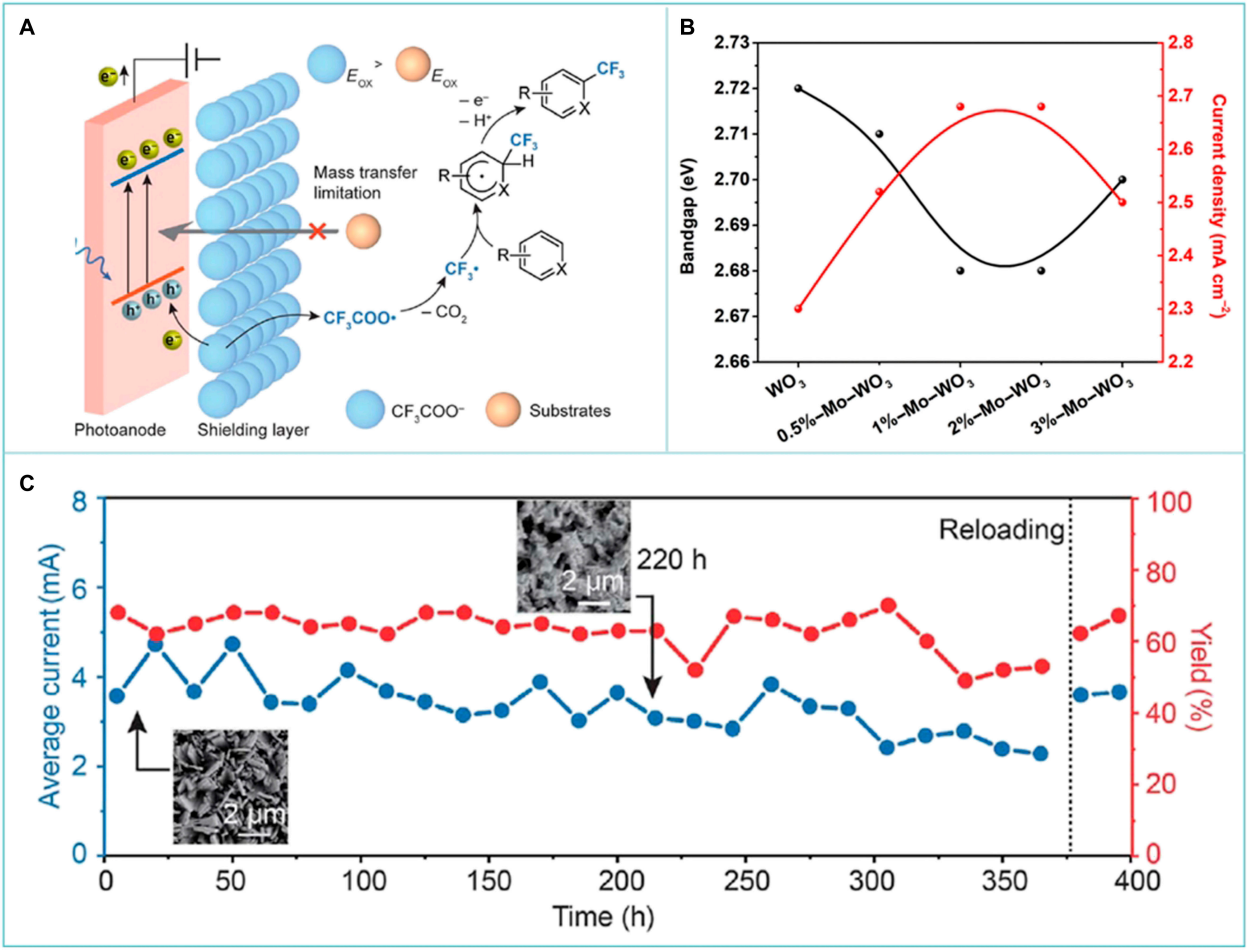
Schematic diagram and performances of photoelectrochemical (PEC) high-value-added chemical production. (A) Schematic illustration of the ion-shielding heterogeneous photoelectrocatalysis strategy. (B) Photocurrent at 1.23 V vs. a saturated calomel electrode (SCE) and bandgap of WO_3_ doped with different amounts of Mo. (C) PEC stability of ion-shielding heterogeneous photoanodes. Reprinted with permission from Chen et al. [5]. Copyright 2024, American Association for the Advancement of Science.

PEC oxidation of small molecules is crucial for alleviating the dependence on fossil fuels. IonShield-hPEC decarboxylative trifluoromethylation provides a distinct strategy for highly effective and selective PEC high-value-added chemical production. The directions of further research are stated below. PEC efficiency could be further enhanced via the modification of photoanodes, such as defect engineering, crystal facet modulation, heterojunction construction, and cocatalyst deposition. Employing single-atom noble metals with well-defined active centers for surface modification of photoanodes is expected to achieve PEC value-added chemicals with high selectivity.
